# Seven versus 14 Day Antibiotic Treatment Duration in Patients with Bacteremia: A Meta-Analysis of Randomized Controlled Trials

**DOI:** 10.1017/ash.2025.278

**Published:** 2025-09-24

**Authors:** Paddy Ssentongo, Cory Hale, Anna Ssentongo, David Ingram

**Affiliations:** 1 Penn State Hershey Medical Center; 2 Penn State Health Milton S. Hershey Medical Center

## Abstract

**Background:** Bacteremia is associated with significant morbidity and mortality. At least 14 days of antibiotic treatment has traditionally been the standard of care. However, shortening the duration of antibiotic therapy is a key strategy for improving antimicrobial stewardship. This meta-analysis of randomized controlled trials (RCTs), including the recently published BALANCE trial, seeks to identify the duration of antibiotics needed to optimize this mortality benefit by comparing seven versus 14 days of antibiotic duration. **Hypothesis:** The mortality risk ratio (RR) in the 7-day group is similar to 14-day group. **Methods:** Multiple electronic databases and trial registries were searched on December 29, 2024, for RCTs reporting mortality outcomes in patients with bacteremia treated for seven versus 14 days of antibiotics. We estimated the effect of these two-treatment durations using random-effects meta-analyses with the generic inverse variance method. Subgroup analyses were conducted to assess the impact of the source of bacteremia on mortality. **Results:** Four eligible RCTs consisting of 4,794 patients with bacteremia, were included. Median age was 71 years (interquartile range (IQR): 69-73), and 47% (IQR: 45%-49%) were male. Of the patients with bacteremia, 87% had gram-negative bacteria and 13% gram-positive bacteria. Patients with Staphylococcus aureus bacteremia, severe immune compromise, prosthetic heart valves, syndromes with well-defined requirement for prolonged treatment such as infective endocarditis or osteomyelitis, single positive blood culture with common contaminant, Candida or other fungi were excluded. Overall mortality rate was 8%. The RR for 90-day and 30-day mortality between 7 versus 14 days was 0.92 (95% CI: 0.79 – 1.06) and 0.92 (95% CI: 0.96-1.12), respectively. Median antibiotic-free days were higher in the 7-day group than 14- day group (19 days vs 14 days, p=0.03). The rates of Clostridioides difficile infection were similar in two groups (1.6% vs 1.5%, p=0.97). Subgroup analysis indicated no effect modification by the source of bacteremia. The risk of bias was assessed as low. **Conclusions:** This systematic review and meta-analysis of RCTs found no difference in mortality between 7- and 14-day treatment durations in low-risk patients with non-Staphylococcus aureus bacteremia. Reducing antibiotic treatment for uncomplicated gram-negative and gram-positive bacteremia to 7 days is a critical antibiotic stewardship intervention.

